# Resveratrol modulates cocaine-induced inhibitory synaptic plasticity in VTA dopamine neurons by inhibiting phosphodiesterases (PDEs)

**DOI:** 10.1038/s41598-017-16034-9

**Published:** 2017-11-15

**Authors:** Yan Li, Laikang Yu, Li Zhao, Fanxing Zeng, Qing-song Liu

**Affiliations:** 1 Department of Exercise Physiology, Beijing Sport University, Beijing, 100084 China; 2 Department of Pharmacology and Toxicology, Medical College of Wisconsin, 8701 Watertown Plank Road, Milwaukee, WI 53226 USA

## Abstract

Resveratrol is a natural phytoalexin synthesized by plants, including grapes. It displays a wide range of neuroprotective benefits associated with anti-aging. Recent studies have shown that resveratrol regulates dopaminergic transmission and behavioral effects of drugs of abuse. The goal of the present study is to investigate whether and how resveratrol alters basal inhibitory synaptic transmission and cocaine-induced inhibitory synaptic plasticity in dopamine neurons of the ventral tegmental area (VTA). We report that resveratrol elevated cAMP levels by itself and further potentiated a forskolin-induced increase in cAMP levels in midbrain slices, consistent with reported effects of inhibition of phosphodiesterases (PDEs). Resveratrol potentiated GABA_A_ and GABA_B_-mediated inhibitory postsynaptic currents (IPSCs) in VTA dopamine neurons, and these effects were mediated by a protein kinase A (PKA)–dependent enhancement of presynaptic GABA release. In addition, we found that resveratrol blocked endocannabinoid-mediated long-term synaptic depression in VTA dopamine neurons. Resveratrol pretreatments attenuated cocaine-induced conditioned place preference and blocked the cocaine-induced reduction of GABAergic inhibition in VTA dopamine neurons. Together, these results provide evidence that resveratrol modulates basal inhibitory synaptic transmission, cocaine-induced synaptic plasticity, and drug-cue associative learning.

## Introduction

Resveratrol (3,4′,5-trihydroxy-trans-stilbene), a constituent of red wine, produces a wide range of health benefits associated with anti-aging, including protection against type 2 diabetes, obesity, cancer, heart disease, and neurodegenerative diseases^1^. Since the discovery that resveratrol mimics the life-span extending effects of calorie restriction in budding yeast^2^, this compound has attracted great interest. However, past research has focused on its role in protecting against aging-related diseases^1^. Recent studies have shown that resveratrol regulates dopaminergic transmission and behavioral effects of drugs of abuse. Acute resveratrol treatment enhances cocaine-induced increases in dopamine D_1_ receptor signaling and locomotor activity in mice, presumably via mechanisms involving the inhibition of monoamine oxidases^3^. In contrast, acute resveratrol treatment is ineffective at altering methamphetamine-induced hyperactivity in mice, while repeated resveratrol treatments decrease methamphetamine-induced hyperactivity in mice and dopamine overflow from rat striatal slices^4^. There are conflicting reports regarding whether resveratrol alters cocaine-induced conditioned place preference (CPP). Resveratrol has been shown to enhance cocaine CPP by activating the NAD(+)-dependent histone deacetylase sirtuins^5^. However, another study has shown that resveratrol is ineffective in altering CPP but attenuates cocaine withdrawal-induced anxiety^6^.

A recent study has identified phosphodiesterases (PDEs) as a direct target for resveratrol, and both resveratrol and the selective PDE4 inhibitor rolipram ameliorate aging-related metabolic phenotypes through inhibition of PDEs^7^. PDEs are a family of enzymes that hydrolyze intracellular cAMP and cGMP^8^. There are 11 subtypes of PDEs (PDE1-11), several of which are expressed in the brain^9,10^. Resveratrol inhibits PDE1, PDE3 and PDE4^7^. PDE1 and PDE3 hydrolyze both cAMP and cGMP, while PDE4 is specific for cAMP^9,10^. Resveratrol raises both cAMP and cGMP levels in Hela cells^7^. Non-selective PDE and PDE4-specific inhibitors reduce drug intake and/or drug seeking for psychostimulants, alcohol, and opioids^11,12^. Selective PDE4 inhibitors such as rolipram significantly reduce cocaine-induced increases in locomotor activity, behavioral sensitization, conditioned place preference (CPP) and self-administration^13–17^. We have shown that rolipram blocks endocannabinoid-mediated long-term depression of inhibitory synaptic transmission (I-LTD) in dopamine neurons of the ventral tegmental area (VTA)^16^ and prevents the repeated cocaine treatment-induced imbalance between excitation and inhibition in VTA dopamine neurons^17^. Although resveratrol has been shown to enhance AMPAR expression via AMP-activated protein kinase-mediated protein translation in cultured neurons^18^, it was unknown whether resveratrol modulates inhibitory synaptic transmission and plasticity. The present study was undertaken to investigate whether resveratrol regulates GABA_A_ and GABA_B_ receptor-mediated inhibitory postsynaptic currents (IPSCs) in VTA dopamine neurons. In addition, we have shown that endocannabinoid-mediated I-LTD is required for the cocaine-induced reduction of GABAergic inhibition to VTA dopamine neurons^19,20^. We therefore examined whether resveratrol modulates I-LTD. Finally, we investigated whether systemic administration of resveratrol altered cocaine-induced CPP and reduction of GABAergic inhibition in VTA dopamine neurons.

## Results

### Resveratrol increased cAMP levels in the VTA

**Figure 1:**
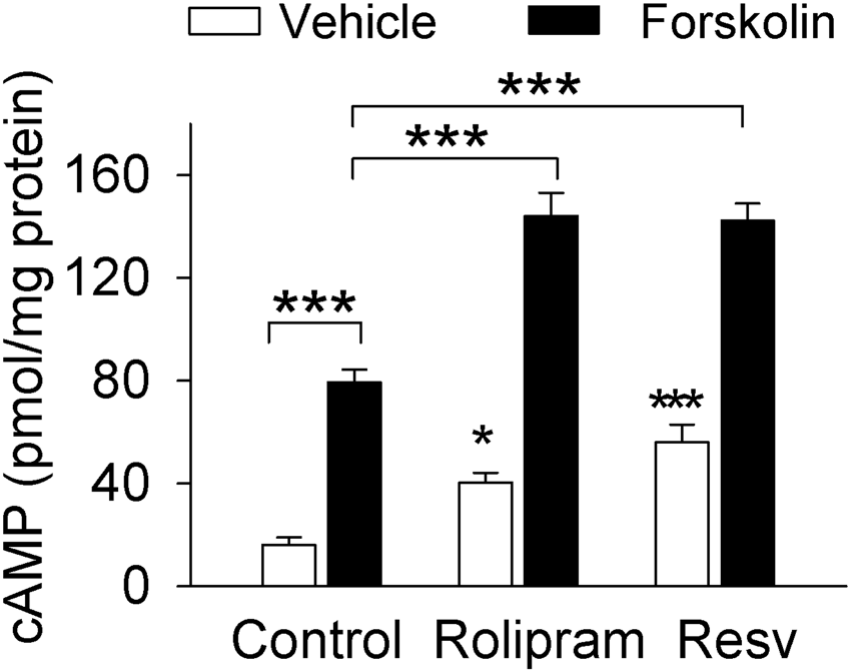
The non-selective PDE inhibitor resveratrol and the PDE4 inhibitor rolipram elevated cAMP levels and further potentiated the forskolin-induced increase in cAMP in midbrain slices. Slices were incubated with vehicle, rolipram (1 µM), resveratrol (Resv, 100 µM) and/or forskolin (10 µM) as indicated in the figure. Resveratrol, rolipram, and forskolin by themselves significantly increased cAMP levels; resveratrol or rolipram further potentiated the forskolin-induced increase in cAMP levels (*p < 0.05 vs. vehicle; ***p < 0.001, n = 8–13). Error bars in this and other figures indicate SEM.

Resveratrol was recently found to be a non-selective PDE inhibitor (inhibition of PDE1, 3, 4)^7^, while rolipram is a selective PDE4 inhibitor. We determined whether resveratrol increased cAMP levels in the VTA. VTA slices were treated with vehicle, resveratrol (100 µM), rolipram (1 µM) and/or the adenylyl cyclase activator forskolin (10 µM) for 30 min. They were then washed, frozen in liquid nitrogen, and homogenized. cAMP levels were measured using an ELISA kit (Enzo). A two-way ANOVA showed that forskolin (*F*_(1,52)_ = 244.05, *p* < 0.001) and the PDE inhibitors (*F*_(2,52)_ = 34.13, *p* < 0.001) significantly increased cAMP levels, and there was a significant interaction between forskolin and PDE inhibitors (*F*_(2, 52)_ = 4.14, *p* = 0.021). Tukey’s *post-hoc* tests indicated that resveratrol (Resv) (*p* < 0.001) or rolipram (*p* < 0.001) significantly increased cAMP levels and further potentiated the forskolin-induced increase in cAMP levels (*p* < 0.001; Fig. 1). The latter finding suggests that resveratrol and forsklin increase cAMP via distinct mechanisms. These results are consistent with previous findings that resveratrol is a PDE inhibitor^7^.

### Resveratrol potentiated GABA_A_ receptor-mediated IPSCs

**Figure 2:**
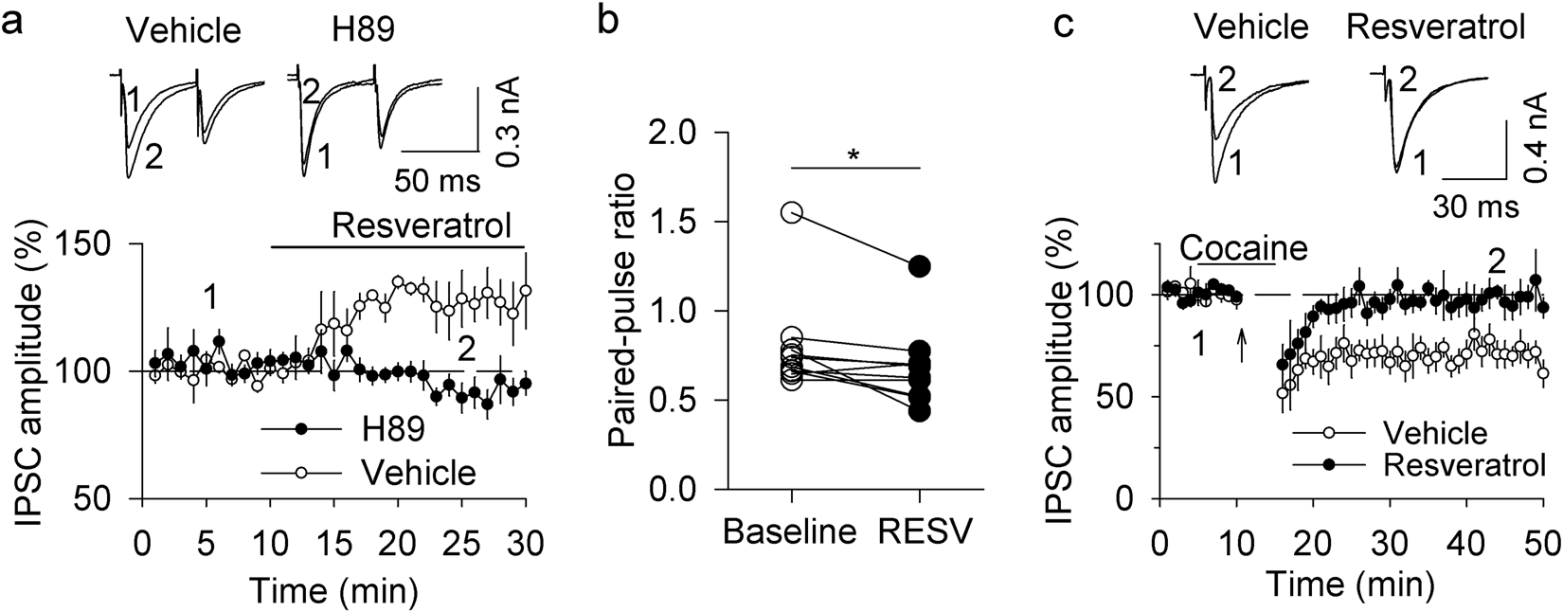
Resveratrol potentiated GABA_A_ receptor-IPSCs by enhancing cAMP/PKA signaling and blocked I-LTD in VTA dopamine neurons. (**a**,**b**). Bath application of resveratrol (100 µM) increased the amplitude of evoked IPSCs (*p* < 0.01, n = 10), which was accompanied by a decrease in the PPR (*p < 0.05, n = 10). This potentiation was blocked by the PKA inhibitor H-89 (10 µM; *p < *0.05 vs. resveratrol alone, n = 6). (**c**) The combination of cocaine application and 10 Hz stimulation for 5 min induced I-LTD in VTA dopamine neurons (*p* < 0.001, n = 7), which was blocked by the continuous presence of resveratrol (100 µM; *p* < 0.01 vs. control, n = 6).

We examined the effects of resveratrol on GABA_A_ receptor-mediated IPSCs in VTA dopamine neurons. To isolate GABA_A_ receptor-mediated IPSCs, the glutamate receptor antagonists CNQX (20 µM) and D-AP5 (50 µM) and the GABA_B_ receptor antagonist CGP 55845 (1 µM) were present in the ACSF throughout the experiments. IPSCs were evoked by paired-pulse stimulation with an inter-pulse interval of 50 ms. Bath application of resveratrol (100 µM) caused a significant increase in the amplitude of IPSCs (129.46 ± 9.49% of baseline, *t*_9_ = 3.018, *p* = 0.015; Fig. 2a). The enhancement of IPSC amplitude was accompanied by a decrease in the paired-pulse ratio (PPR) (*t*_9_ = 2.747, *p* = 0.023; Fig. 2b). The PPR was calculated as the ratio of the amplitude of the second IPSCs to that of the first IPSCs. The decrease in the PPR suggests an increase in the probability of presynaptic GABA release^21^. Bath application of resveratrol at a low concentration (10 µM) had no significant effect on the amplitude of IPSCs (98.01 ± 2.17% of baseline, *t*_5_ = 1.077, *p* = 0.331) and the PPR (*t*_5_ = 0.394, *p* = 0.710; Supplementary Fig. S1). One possibility is that PDE expression in the VTA may be low, perhaps requiring higher concentrations of resveratrol to affect IPSCs.

It has been shown that activating cAMP/PKA signaling enhances neurotransmitter release at many excitatory and inhibitory synapses^22–25^. Next, we examined whether PKA was involved in the resveratrol-induced increase in IPSC amplitude. The PKA inhibitor H-89 (10 µM) was present in the ACSF throughout the experiment. The effects of resveratrol on IPSCs were blocked in the continuous presence of H-89 (93.08 ± 5.67% of baseline, *t*_14_ = 2.768*, p* = 0.015 vs. resveratrol alone; Fig. 2a). These results suggest resveratrol potentiates GABA_A_-mediated IPSCs via an enhancement of cAMP/PKA signaling.

Resveratrol is a competitive, reversible inhibitor of PDEs^7^. We tested whether the resveratrol-induced enhancement of GABA_A_-IPSCs could be reversed upon washout. After stable baseline recordings of IPSCs for 10 min, resveratrol (100 µM) was applied for 20 min, which was followed by a 20 min washout. Consistent with the earlier observation in Fig. 2, we found that resveratrol application enhanced IPSCs (135.88 ± 7.68% of baseline, *t*_6_ = 7.678*, p < *0.001; Supplementary Fig. S2). However, the potentiation of IPSCs was not reversed after 20 min of washout (139.72 ± 7.19% of baseline, *t*_6_ = 1.876*, p* = 0.110 vs. resveratrol application; Supplementary Fig. S2). Our past experience indicates that pharmacological effects of many lipophilic compounds such as cannabinoid CB_1_ receptor agonists and antagonists are not reversible due to poor washout from brain slices^19^. The difficulty to wash out resveratrol from brain slices may explain why the effect of resveratrol on IPSCs was not reversed during the time window tested.

### Resveratrol blocked I-LTD in VTA dopamine neurons

We have shown that a pathophysiologically relevant concentration of cocaine (3 µM) enables subthreshold stimulation to induce I-LTD in VTA dopamine neurons of midbrain slices^19^, and that such I-LTD is mediated by activation of the D_2_ receptor and the CB_1_ receptor, followed by subsequent inhibition of cAMP/PKA signaling^20,26^. Having shown that resveratrol enhanced the amplitude of GABA_A_ receptor-mediated IPSCs, we next examined whether resveratrol affected I-LTD in VTA dopamine neurons. Whole-cell voltage-clamp recordings (holding potential −70 mV) were made from VTA dopamine neurons. IPSCs were evoked by stimulating inhibitory synaptic afferents at 0.1 Hz. The AMPA receptor antagonist CNQX (20 µM), the NMDA receptor antagonist AP-5 (50 µM), and the GABA_B_ receptor antagonist CGP 55845 (1 µM) were present in the ACSF throughout the experiments. Consistent with our previous studies^16,19,20^, we found that repeated synaptic stimulation (10 Hz, 5 min) in the presence of a low concentration of cocaine (3 µM) induced I-LTD (73.12 ± 5.34% of baseline, *t*_12_ = 5.069, *p* < 0.001; Fig. 2c). This I-LTD was blocked by the continuous presence of resveratrol (100 µM) (95.89 ± 3.40% of baseline, *t*_11_ = 3.456, *p* = 0.005 vs. control; Fig. 2c). These results indicate that resveratrol blocked I-LTD induction in VTA dopamine neurons.

### Resveratrol potentiated GABA_B_ receptor-mediated IPSCs

**Figure 3:**
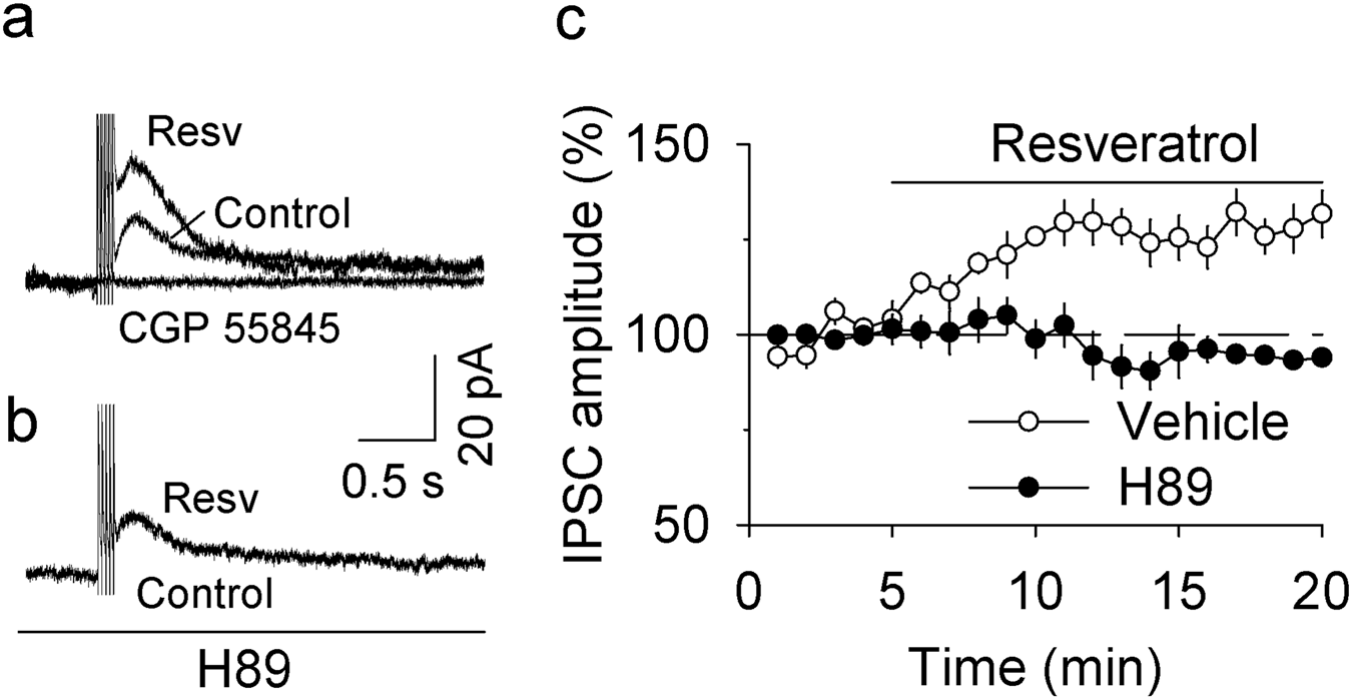
Resveratrol potentiated GABA_B_ receptor-IPSCs by enhancing cAMP/PKA signaling. (**a**) Bath application of resveratrol (Resv, 100 µM) increased the amplitude of GABA_B_ receptor-IPSCs (*p* < 0.001, n = 5), which was blocked by the selective GABA_B_ receptor antagonist CGP 55845 (1 μM; *p* < 0.001, n = 4). (**b**) The resveratrol-induced potentiation of GABA_B_ receptor-IPSCs was blocked by the PKA inhibitor H-89 (10 µM; *p < *0.001 vs. resveratrol alone, n = 5). (**c**) Time course of the effects of resveratrol on GABA_B_ receptor-IPSCs in the presence and absence of the PKA inhibitor H-89.

GABA_B_ receptors are linked to G protein-gated inwardly-rectifying potassium (GIRK) channels^27^. Repetitive, short-burst electrical stimulation induces GABA_B_ receptor-mediated slow IPSCs in VTA dopamine neurons^28^. Next, we examined the effect of resveratrol on GABA_B_ receptor-mediated IPSCs in VTA dopamine neurons. Whole-cell recordings were made from VTA dopamine neurons at a holding potential of −55 mV. GABA_B_ receptor-mediated IPSCs were evoked with five stimuli (0.3 ms) at 50 Hz and were isolated pharmacologically with GABA_A_ receptor blocker picrotoxin (100 μM), AMPAR antagonist CNQX (10 μM), NMDAR blocker MK-801 (10 μM), D_2_ dopamine receptor antagonist sulpiride (1 μM), and group I mGluR antagonist CPCCOEt (100 µM) in the ACSF. We found that bath application of resveratrol (100 μM) enhanced the amplitude of GABA_B_ receptor-mediated IPSCs in VTA dopamine neurons (128.00 ± 5.70% of baseline, *t*_8_ = 9.311*, p* < 0.001 vs. baseline; Fig. 3a,c). The IPSCs were blocked by the selective GABA_B_ receptor antagonist CGP 55845 (1 μM) (Fig. 3a), indicating that they are indeed GABA_B_-mediated IPSCs. In the presence of the PKA inhibitor H89 (10 µM), bath application of resveratrol (100 µM) did not significantly alter GABA_B_-mediated IPSCs (94.47 ± 1.72% of baseline, *t*_8_ = 18.42*, p* < 0.001 vs. resveratrol alone; Fig. 3b,c). Thus, resveratrol potentiated both GABA_A_- and GABA_B_-mediated IPSCs via cAMP/PKA-dependent mechanisms.

### The effects of resveratrol pretreatments on the acquisition of cocaine CPP

**Figure 4:**
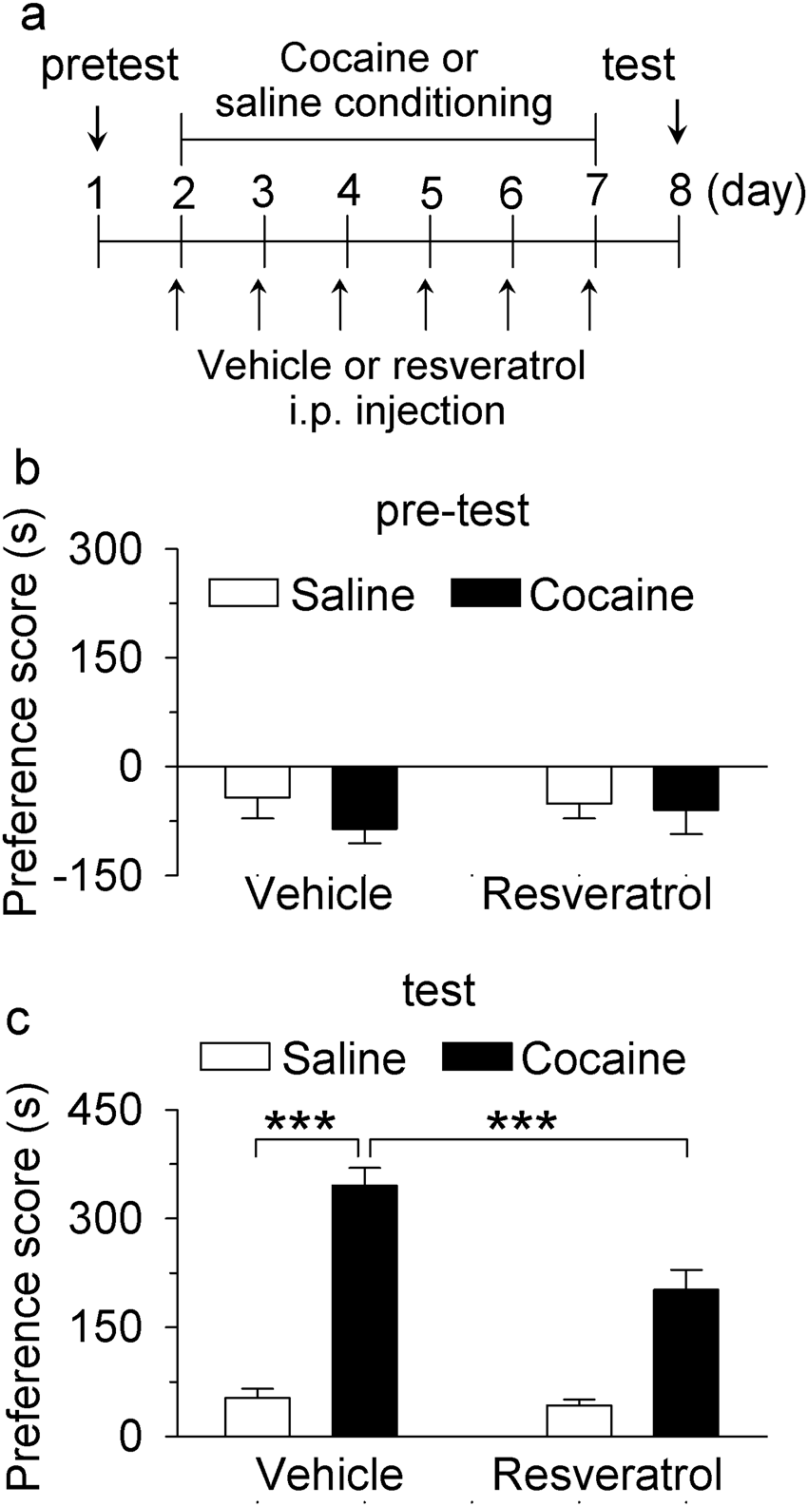
Resveratrol pretreatments during the conditioning phase attenuated the acquisition of CPP to cocaine. (**a**) Timeline of drug treatment and behavioral paradigm. Groups of mice received pre-tests on day 1 for unconditioned place preference (baseline bias). Then the mice received saline and cocaine place conditioning once daily for 6 days. CPP testing was carried out on the eighth day. Resveratrol (20 mg/kg) was i.p. injected 30 min prior to each saline or cocaine pairing. (**b**) Pre-test indicates that mice did not exhibit significant baseline bias in place preference in all groups (*p* > 0.05, n = 6–8/group). (**c**) Resveratrol pretreatments significantly attenuated CPP in cocaine-conditioned mice but did not affect CPP scores in saline-conditioned mice (**p < 0.01, ***p < 0.001, n = 6–8/group).

We have shown that pretreatments with the selective PDE4 inhibitor roplipram 30 min before place conditioning attenuates cocaine CPP in rats^16^ and cocaine-induced locomotor sensitization in mice^17^. We examined whether pretreatments with resveratrol before place conditioning affected the acquisition of cocaine CPP. Mice underwent cocaine or saline conditioning as described in Materials and Methods and the timeline of the experimental design is shown in Fig. 4a. Mice did not show unconditioned place preference in the pre-test (*p* > 0.05, Fig. 4b). Resveratrol (20 mg/kg, i.p.) or vehicle was i.p. injected 30 min before each cocaine or saline pairing on all conditioning days. We found that cocaine (15 mg/kg) conditioning (*F*_(1,24)_ = 120.32, *p* < 0.001) and resveratrol pretreatments (*F*_(1,24)_ = 13.96, *p* = 0.001) significantly altered the preference score, and there was a significant interaction between cocaine conditioning and resveratrol pretreatments (*F*_(1,24)_ = 10.48, *p* = 0.004). Tukey’s *post hoc* tests indicated that cocaine conditioning induced CPP in vehicle-pretreated mice (*p* < 0.001), while resveratrol pretreatments significantly attenuated cocaine CPP (*p* < 0.001; Fig. 4c).

### Resveratrol pretreatments blocked the reduction of GABAergic inhibition induced by cocaine conditioning

**Figure 5:**
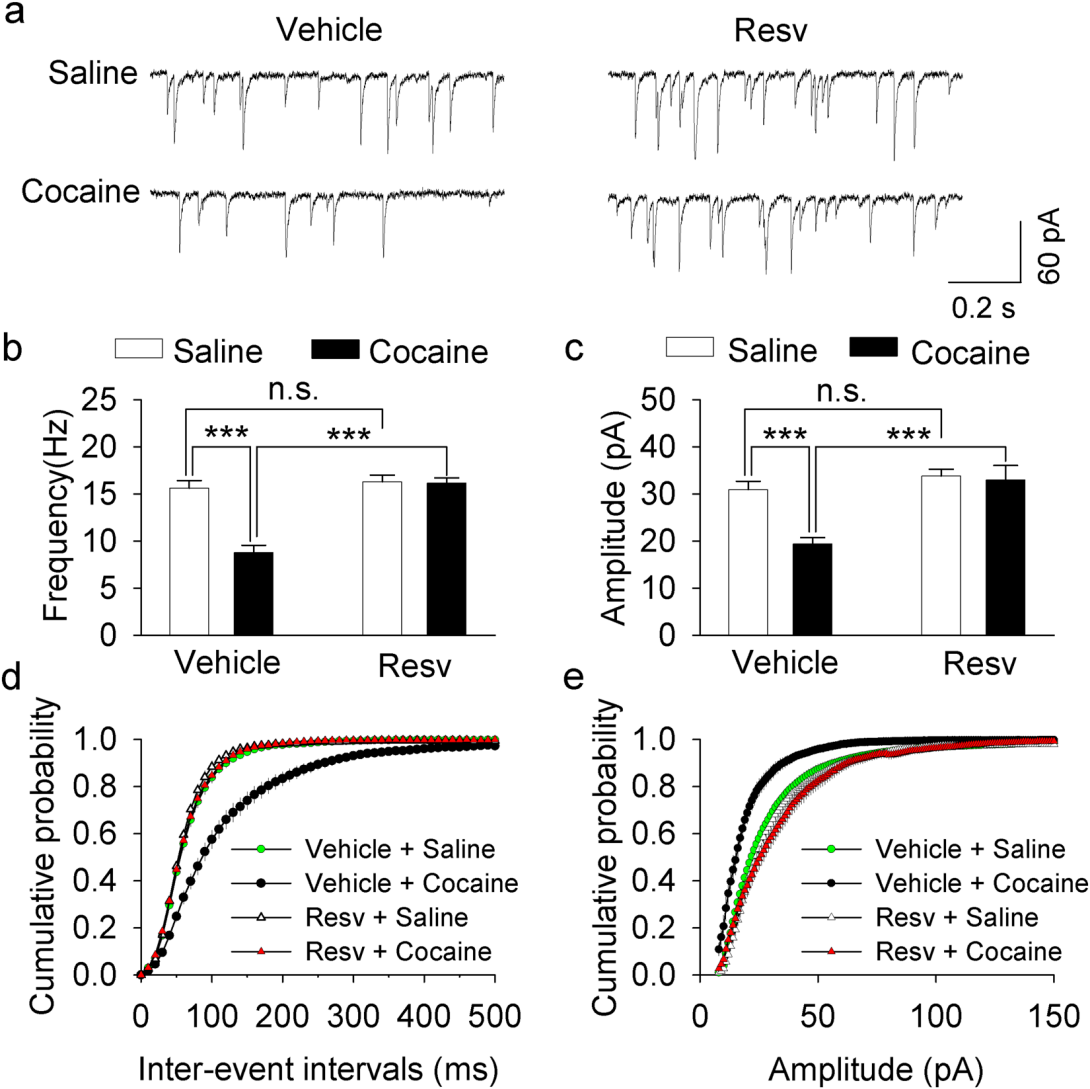
Resveratrol pretreatments blocked the reduction of GABAergic inhibition to dopamine neurons induced by cocaine conditioning. (**a**) Representative sIPSCs recorded from VTA dopamine neurons in slices prepared from saline- or cocaine-conditioned mice pre-treated with vehicle or resveratrol (Resv). (**b**,**c**) The average frequency (**b**) and amplitude (**c**) of sIPSCs in VTA dopamine neurons in these four groups of mice. The mean frequency and amplitude of sIPSCs were significantly decreased in cocaine-conditioned, vehicle-treated mice (***p < 0.001, n = 12–13), and this decrease was blocked by resveratrol pretreatments (***p < 0.001, n = 12–13). (**d**,**e**) Cumulative probability plots indicate that cocaine exposure led to shifts in the distribution of the inter-event intervals (**d**) and amplitude (**e**) in vehicle-treated mice; these shifts were blocked by resveratrol pretreatments (*p* < 0.001, n = 12–13).

We and others have shown that cocaine exposure *in vivo* reduces the strength of GABAergic inhibition to VTA dopamine neurons^19,29,30^. The selective PDE4 inhibitor rolipram blocks the cocaine-induced reduction in the mean frequency and amplitude of spontaneous IPSCs (sIPSCs) in VTA dopamine neurons^17^. Having shown that pretreatment with resveratrol attenuated cocaine CPP, we next investigated whether cocaine CPP was associated with changes in sIPSCs and whether resveratrol pretreatments altered cocaine-induced effects on sIPSCs. One day after the CPP test shown in Fig. 4, the mice were sacrificed and midbrain slices were prepared. sIPSCs were recorded from VTA dopamine neurons in these four groups of mice. Two-way ANOVA revealed that cocaine conditioning and resveratrol pretreatments had significant effects on the mean frequency of sIPSCs (cocaine: *F*_(1,49)_ = 25.488, *p* < 0.001; resveratrol: *F*_(1,49)_ = 33.455, *p* < 0.001; cocaine × resveratrol interaction: *F*_(1,49)_ = 16.280, *p* < 0.001; Fig. 5a,b), and the mean amplitude of sIPSCs (cocaine: *F*_(1,49)_ = 10.573, *p* = 0.002; resveratrol: *F*_(1,49)_ = 19.053, *p* < 0.001; cocaine × resveratrol interaction: *F*_(1,49)_ = 8.183, *p* = 0.006; Fig. 5a,c). Tukey’s *post hoc* tests indicated that cocaine conditioning led to significant decreases in the mean frequency (*p* < 0.001; Fig. 5b) and amplitude of sIPSCs (*p* < 0.001; Fig. 5c), which was blocked by resveratrol pretreatment (*p* < 0.001; Fig. 5b,c). The cumulative distribution for inter-event intervals of sIPSCs was shifted to the right (i.e., longer interval and less frequent) in the vehicle/cocaine group (*p* < 0.001; Fig. 5d), and this shift was blocked by resveratrol pretreatments (*p < *0.001; Fig. 5d). The cumulative distribution for the amplitude of sIPSCs was shifted to the left (i.e., smaller value) in the vehicle/cocaine group (*p* < 0.001; Fig. 5e), and this shift was blocked by resveratrol pretreatments (*p* < 0.001; Fig. 5e). Together, these results indicate that cocaine conditioning led to a reduction of the frequency and amplitude of sIPSCs, and that this reduction was blocked by resveratrol pretreatments.

## Discussion

The present study has shown that resveratrol increased cAMP levels in midbrain slices. In addition, we have shown that resveratrol potentiated both GABA_A_- and GABA_B_-mediated IPSCs via cAMP/PKA-dependent mechanisms, and blocked I-LTD in VTA dopamine neurons. Further, resveratrol attenuated cocaine CPP and the cocaine conditioning-induced decrease in sIPSCs in VTA dopamine neurons. Together, these results suggest that resveratrol attenuates cocaine-induced inhibitory synaptic plasticity and rewarding effects.

A recent study has shown that resveratrol raises both cAMP and cGMP levels in Hela cells via inhibition of PDE1, PDE3 and PDE4^7^. To determine whether resveratrol has similar effects in the brain, we determined whether it enhanced cAMP levels in midbrain slices. The slices were incubated with vehicle, resveratrol, rolipram alone or in combination with the adenylyl cyclase activator FSK, and cAMP levels in midbrain slices were measured using an ELISA kit. We found that resveratrol, rolipram, and forskolin by themselves increased cAMP levels compared with that of vehicle, and that resveratrol or rolipram potentiated the forskolin-induced increase in cAMP. The latter finding is consistent with the idea that resveratrol and rolipram are PDE inhibitors, but not adenylyl cyclase activators^7^.

Bath application of resveratrol caused a significant increase in the amplitude of evoked GABA_A_-mediated IPSCs, which was accompanied by a decrease in the PPR. A change in the PPR suggests a change in presynaptic neurotransmitter release probability^21^. These results suggest that resveratrol induced an increase in GABA release in the VTA. The effect of resveratrol on GABA_A_-IPSCs was blocked by the PKA inhibitor H89. Drugs that enhance cAMP/PKA signaling enhance glutamate or GABA release at central synapses^22–25^, whereas drugs that inhibit cAMP/PKA signaling decrease neurotransmitter release^31,32^. Thus, resveratrol enhances GABA_A_-mediated IPSCs through a PKA-dependent potentiation of presynaptic GABA release. We have shown previously that the combination of 10 Hz stimulation with a low concentration of cocaine induces endocannabinoid-mediated I-LTD in VTA dopamine neurons^19^, and this I-LTD is dependent on cAMP/PKA signaling^20^. The present study indicates that resveratrol blocked I-LTD in VTA dopamine neurons. Since a decrease in presynaptic cAMP/PKA activity is required for I-LTD induction^20,26^, it is likely that resveratrol blocked I-LTD via an enhancement of presynaptic cAMP/PKA signaling.

**Figure 6:**
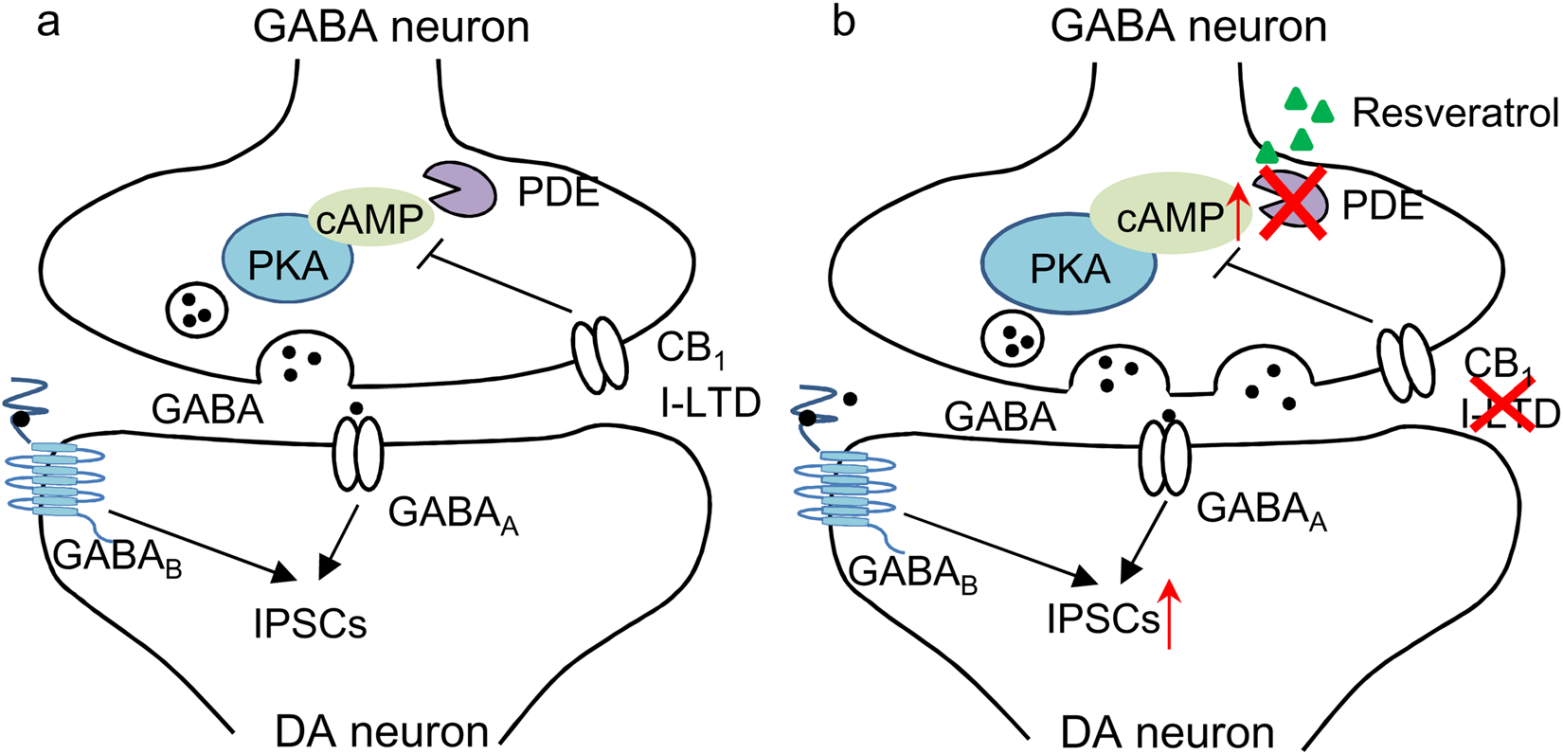
Working model of resveratrol-induced modulation of IPSCs and I-LTD in the VTA. (**a**) In this model, PDEs degrade cAMP in VTA GABA neurons, leading to suppression of PKA activation of GABA release. GABA activates synaptic GABA_A_ receptors and perisynaptic GABA_B_ receptors to produce GABA_A_- and GABA_B_-IPSCs. Activation of the Gα_*i/o*_-coupled CB_1_ receptor also decreases cAMP/PKA signaling in presynaptic GABA axonal terminals, leading to I-LTD. (**b**) The non-selective PDE inhibitor resveratrol increases cAMP/PKA signaling by inhibiting cAMP degradation. As a result, GABA_A_- and GABA_B_-IPSCs are enhanced, and CB_1_ receptor-mediated I-LTD is blocked. DA neuron means dopamine neuron.

While GABA_A_-mediated IPSCs can be induced by single synaptic stimulation, GABA_B_-mediated IPSCs often require repetitive synaptic stimulation at high-frequency^27^. GABA_B_ receptors are located at perisynaptic sites and high-frequency stimulation causes spillover of GABA to activate these receptors^27^. Resveratrol also produced an enhancement of GABA_B_-mediated IPSCs in VTA dopamine neurons, and this effect was blocked by the PKA inhibitor H89. The similar enhancement of GABA_A_- and GABA_B_-mediated IPSCs further suggests that resveratrol enhances presynaptic GABA release in VTA dopamine neurons (Fig. 6). Genetic deletion of GABA_B_ receptors from dopamine neurons in adult mice increased cocaine-induced locomotion^33^. GABA_B_ agonists and positive allosteric modulators have been shown to reduce behavioral effects of drugs of abuse including cocaine^34^. Enhancing GABA_B_ receptor-mediated inhibition may also contribute to the inhibitory effects of resveratrol on cocaine CPP (see below).

The effects of resveratrol on IPSCs should depend on where the PDEs are expressed. The distribution of PDE4 in the brain has been well-studied relative to other types of PDEs. PDE4 has four isoforms: PDE4A, PDE4B, PDE4C, and PDE4D^35,36^. PDE4A, PDE4B, and PDE4D are the main isoforms expressed in the rodent brain^37,38^. PDE4A is the most abundant isoform expressed in the VTA, but its expression in different cell types of the VTA has not been studied^38^. We suspect that resveratrol inhibits PDE4A on VTA GABA neurons, leading to an enhancement of GABA release.

PDE inhibitors reduce drug intake and/or drug seeking for psychostimulants, alcohol, and opioids^11,12^. The selective PDE4 inhibitor rolipram significantly reduces cocaine-induced increases in locomotor activity, behavioral sensitization, CPP and self-administration^13–17^. The present study has shown that pretreatments with resveratrol attenuated cocaine CPP. This finding stands in contrast with two previous studies. Resveratrol has been shown to enhance cocaine CPP in mice by activating sirtuins^5^. However, another study has shown that it is ineffective in altering CPP in rats^6^. The dose (20 mg/kg, i.p.) of resveratrol used here was the same as that used in the mouse study^5^, while doses of 20–110 mg/kg were used in the rat study^6^. The reason for the discrepancy among these studies is not yet clear but could not be attributable to the doses used.

In addition, we found that resveratrol pretreatments blocked the cocaine conditioning-induced reduction of GABAergic inhibition in VTA dopamine neurons. Cocaine conditioning led to decreases in the frequency and amplitude of sIPSCs, and these decreases were blocked in mice that received resveratrol pretreatments. Our previous studies suggest that endocannabinoid-mediated I-LTD provides a putative mechanism for the cocaine-induced reduction of GABAergic inhibition in VTA dopamine neurons^19^. Resveratrol may block the cocaine-induced reduction of GABAergic inhibition via a mechanism of I-LTD blockade.

In summary, we have shown that resveratrol enhances GABA_A_- and GABA_B_-mediated IPSCs in VTA dopamine neurons. Additionally, it blocked endocannabinoid-mediated I-LTD. Finally, we showed that resveratrol attenuated cocaine CPP and abolished the cocaine-induced reduction of GABAergic inhibition. These results provide evidence that resveratrol blocks cocaine-induced synaptic plasticity in VTA dopamine neurons and drug-cue associative learning.

## Methods and Materials

### Animals

Male C57BL/6J mice (8–10 weeks old) were used for brain slice electrophysiological recordings, behavior experiments and enzyme-linked immunosorbent assay (ELISA). Animal maintenance and use were in accordance with protocols approved by the Institutional Animal Care and Use Committee of the Medical College of Wisconsin.

### Brain slice preparation

Midbrain slices (200 μm) from male C57BL/6J mice were prepared as described previously^39,40^. Mice were anaesthetized by isoflurane inhalation and decapitated. The whole brain was quickly removed and embedded in 3% low-melting-point agarose. Horizontal midbrain slices (200 μm thick) were cut using a vibrating slicer (Leica VT1200s, Nussloch, Germany), using choline-based solution containing (in mM): 110 choline chloride, 2.5 KCl, 1.25 NaH_2_PO_4_, 0.5 CaCl_2_, 7 MgSO_4_, 23 NaHCO_3_, 25 glucose, 11.6 sodium ascorbate, and 3.1 sodium pyruvate at room temperature. The slices containing the ventral tegmental area of the midbrain (VTA) were incubated in the sucrose-based solution containing (in mM): 78 NaCl, 68 sucrose, 23 NaHCO_3_, 2.5 KCl, 1.25 NaH_2_PO_4_, 2 CaCl_2_, 2 MgCl_2_ and 25 glucose for 30–40 minutes at room temperature. Then, the slices were allowed to recover in the ACSF containing (in mM): 119 NaCl, 2.5 KCl, 2.5 CaCl_2_, 1 MgCl_2_, 1.25 NaH_2_PO_4_, 23 NaHCO_3_, and 10 glucose.

### Electrophysiological recordings

Whole-cell patch-clamp recording was made on VTA dopamine neurons using patch clamp amplifiers (Multiclamp 700B) under infrared-differential interference contrast (DIC) microscopy. Data acquisition and analysis were performed using DigiData 1440 A or DigiData 1550B digitizers and analysis software pClamp 10 (Molecular Devices). Signals were filtered at 2 kHz and sampled at 10 kHz. Dopamine neurons in the VTA were identified by long duration (>1.5 ms) of spontaneous action potentials in cell-attached configuration^41^ and the presence of large I_h_ currents, rhythmic firing at low frequency and prominent afterhyperpolarization in whole-cell mode^29,42,43^. For recording evoked inhibitory postsynaptic currents (IPSCs), electrical stimulation was delivered by a bipolar tungsten stimulation electrode (WPI) that was placed at fixed distance (~150 μm) rostral to the soma of recorded dopamine neuron. For recordings of GABA_A_ receptor-mediated IPSCs and spontaneous IPSCs, glass pipettes (3–5 MΩ) were filled with an internal solution containing (in mM): 90 K-gluconate, 50 KCl, 10 HEPES, 0.2 EGTA, 2 MgCl_2_, 4 Mg-ATP, 0.3 Na_2_GTP, and 10 Na_2_-phosphocreatine (pH 7.2 with KOH). To isolate GABA_A_ receptor-mediated IPSCs, CNQX (20 µM), D-AP5 (50 µM) and CGP 55845 (1 µM) were present in the ACSF to block AMPA receptors, NMDA receptors and GABA_B_ receptors, respectively. For recording GABA_B_ receptor-mediated IPSCs, glass pipettes (3–5 MΩ) were filled with an internal solution containing (in mM): 140 K-gluconate, 5 KCl, 10 HEPES, 0.2 EGTA, 2 MgCl_2_, 4 Mg-ATP, 0.3 Na_2_GTP, and 10 Na_2_-phosphocreatine (pH 7.2 with KOH). Neurons were voltage-clamped at −70 mV for GABA_A_-IPSCs and −55 mV for GABA_B_-IPSCs. The GABA_B_ receptor-mediated IPSCs were evoked by five stimuli (0.3 ms) at 50 Hz. To isolate GABA_B_ receptor-mediated IPSCs, picrotoxin (100 μM), CNQX (10 μM), MK-801 (10 μM), sulpiride (1 μM), and CPCCOEt (100 µM) were added in the ACSF to block GABA_A_ receptors, AMPA receptors, NMDA receptors and group I metabotropic glutamate receptors (mGluRs), respectively. Series resistance (15–30 MΩ) was monitored throughout all recordings, and data were discarded if the resistance changed by more than 20%. All recordings were performed at 32 ± 1 °C by using an automatic temperature controller (Warner Instruments, Inc).

### The cAMP assay

cAMP levels were measured with the mouse cAMP ELISA kit (Enzo Life Sciences), according to the manufacturer’s protocol. Briefly, horizontal midbrain slices containing the VTA were prepared as described above. Slices were treated with vehicle, resveratrol (100 µM), rolipram (1 µM) and/or the adenylyl cyclase activator forskolin (10 µM) for 30 min, washed, frozen in liquid nitrogen and then homogenized in ice-cold 0.1 M HCl. The homogenates were centrifuged at 13, 000 × g for 50 min at 4 °C to pellet debris. The supernatants were collected for ELISA assay. Absorbance was read at 405 nm with an ELX800 Universal Microplate Reader (Bio-TEK Instruments). Finally, the cAMP level was normalized to the total protein concentration, which was assayed using a BCA method.

### Conditioned place preference (CPP)

CPP experiments were performed based on previously published procedures^16,44^ with minor modifications. The CPP protocol consisted of the following three sections: (1) pre-test (day 1): mice were placed in the middle chamber of the three-chamber conditioning apparatus (Med Associates, St Albans, Vermont) and allowed to explore three chambers freely for 20 min, with time spent in each chamber recorded. Mice showing unconditioned side preference (> = 180 s disparity) were excluded. (2) Conditioning (day 2–7): *Cocaine conditioning*. On days 2, 4 and 6, mice were injected with cocaine (15 mg/kg, i.p.) and confined to one chamber for 30 min; On day 3, 5 and 7, mice were injected with saline (0.9% NaCl, 2 ml/kg, i.p.) and confined to one chamber for 30 min. *Saline conditioning*. Mice were injected with saline (0.9% NaCl, 2 ml/kg, i.p.) daily and were confined to one chamber for 30 min on days 3, 5 and 7 and were confined to the opposite chamber for 30 min on days 2, 4 and 6. (3) CPP test (day 8): All of the mice were allowed to explore freely for 20 min between the two sides, and time spent on each side was recorded. Subgroups of mice also received i.p. infusions of vehicle (5% DMSO in saline) or resveratrol (20 mg/kg) 30 min before each cocaine or saline injections.

### Statistics

Data are present as mean ± SEM. The magnitude of I-LTD and evoked IPSCs was calculated as we have described previously^19,45^. The frequency and amplitude of sIPSCs were analyzed using Minianalysis (Synaptosoft Inc.) as we have described^17^. The paired-pulse ration (PPR) was calculated as the ratio of the amplitude of the second IPSCs to that of the first IPSCs. CPP scores were calculated as the time spent in the cocaine-paired chamber minus the time spent in the saline-paired chamber. Data sets were compared with either Student’s *t*-test (I-LTD, evoked IPSCs amplitude and PPR) or two-way ANOVA (the frequency and amplitude of sIPSCs and CPP scores) followed by Tukey *post hoc* analysis. Paired *t*-test was used for before and after comparison from the same cells, while *t*-test was used for different data set comparison. Results were considered to be significant at *p* < 0.05.

## Electronic supplementary material

Supplementary figures
