# Supplementary material for: Resveratrol modulates cocaine-induced inhibitory synaptic plasticity in VTA dopamine neurons by inhibiting phosphodiesterases (PDEs)
Source: Sci Rep. 2017 Nov 15;7:15657. doi: 10.1038/s41598-017-16034-9 (PMC5688096; doi:10.1038/s41598-017-16034-9)
Supplement: Supplementary file 1 — Supplementary figures [file 41598_2017_16034_MOESM1_ESM.pdf]

Resveratrol modulates cocaine-induced inhibitory synaptic plasticity in VTA dopamine neurons by inhibiting phosphodiesterases (PDEs)

Yan Li[1,2#], Laikang Yu[1,2#], Li Zhao[1]*, Fanxing Zeng[1], and Qing-song Liu[2]*

[1] Department of Exercise Physiology, Beijing Sport University, Beijing 100084, China

[2] Department of Pharmacology and Toxicology, Medical College of Wisconsin, 8701 Watertown Plank Road, Milwaukee, WI 53226, USA

[#] Y.L. and L.Y. contributed equally to this work.

*Correspondence should be sent to:

Qing-song Liu, Ph.D., Department of Pharmacology and Toxicology, Medical College of Wisconsin, 8701 Watertown Plank Road, Milwaukee, WI 53226, USA

Email: qsliu@mcw.edu

Li Zhao, Ph.D., Department of Exercise Physiology, Beijing Sport University, Beijing 100084, China

Email: zhaolispring@126.com

Supplementary Materials

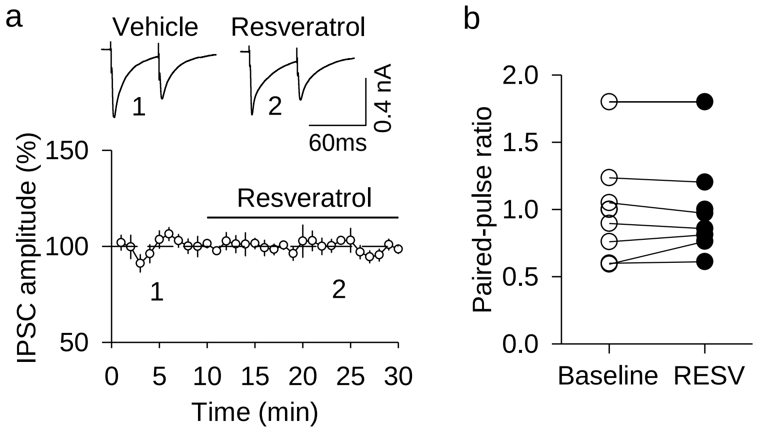

Supplementary Figure S1. Resveratrol at 10 µM had no significant effect on $GABA_A$ receptor-IPSCs in VTA dopamine neurons. **a, b.** Bath application of resveratrol (10 µM) did not alter the amplitude (**a**) and PPR (**b**) of IPSCs (p = 0.331, n = 6).

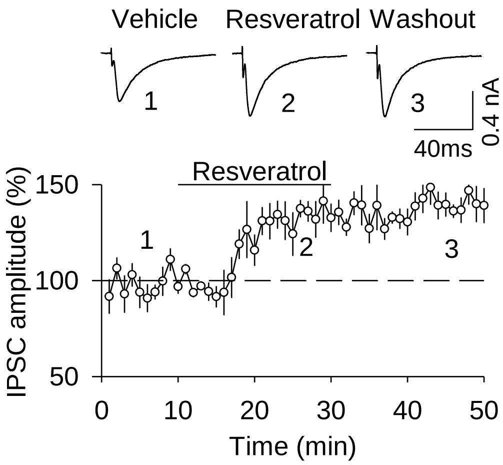

Supplementary Figure S2. The resveratrol-induced enhancement of IPSCs was not reversed during a 20 min washout period. Resveratrol (100 µM) caused an increase in the amplitude of IPSCs ($p < 0.001$, n = 7). However, the potentiation was not reversed upon washout of resveratrol for 20 min ($p = 0.110$ vs. resveratrol application, n = 7).
